# Problematic meta-analyses: Bayesian and frequentist perspectives on combining randomized controlled trials and non-randomized studies

**DOI:** 10.1186/s12874-024-02215-4

**Published:** 2024-04-27

**Authors:** John L. Moran, Ariel Linden

**Affiliations:** 1 The Queen Elizabeth Hospital, Woodville, SA 5011 Australia; 2 Department of Medicine, School of Medicine, University of California, San Francisco, USA

**Keywords:** Meta-analysis, Frequentist, Bayesian, Bayes factors, Posterior probability

## Abstract

**Purpose:**

In the literature, the propriety of the meta-analytic treatment-effect produced by combining randomized controlled trials (RCT) and non-randomized studies (NRS) is questioned, given the inherent confounding in NRS that may bias the meta-analysis. The current study compared an implicitly principled pooled Bayesian meta-analytic treatment-effect with that of frequentist pooling of RCT and NRS to determine how well each approach handled the NRS bias.

**Materials & methods:**

Binary outcome Critical-Care meta-analyses, reflecting the importance of such outcomes in Critical-Care practice, combining RCT and NRS were identified electronically. Bayesian pooled treatment-effect and 95% credible-intervals (BCrI), posterior model probabilities indicating model plausibility and Bayes-factors (BF) were estimated using an informative heavy-tailed heterogeneity prior (half-Cauchy). Preference for pooling of RCT and NRS was indicated for Bayes-factors > 3 or < 0.333 for the converse. All pooled frequentist treatment-effects and 95% confidence intervals (FCI) were re-estimated using the popular DerSimonian-Laird (DSL) random effects model.

**Results:**

Fifty meta-analyses were identified (2009–2021), reporting pooled estimates in 44; 29 were pharmaceutical-therapeutic and 21 were non-pharmaceutical therapeutic. Re-computed pooled DSL FCI excluded the null (OR or RR = 1) in 86% (43/50). In 18 meta-analyses there was an agreement between FCI and BCrI in excluding the null. In 23 meta-analyses where FCI excluded the null, BCrI embraced the null. BF supported a pooled model in 27 meta-analyses and separate models in 4. The highest density of the posterior model probabilities for 0.333 < Bayes factor < 1 was 0.8.

**Conclusions:**

In the current meta-analytic cohort, an integrated and multifaceted Bayesian approach gave support to including NRS in a pooled-estimate model. Conversely, caution should attend the reporting of naïve frequentist pooled, RCT and NRS, meta-analytic treatment effects.

**Supplementary Information:**

The online version contains supplementary material available at 10.1186/s12874-024-02215-4.

## Introduction

The combination of randomized controlled trials (RCT) and non-randomized studies (NRS [1, 2]) within a meta-analysis, that is, using “all” the available information [3–5], has been a problematic exercise both theoretically and practically [1, 2, 6]. With respect to the theoretic, the conventional frequentist analytic approach to such meta-analyses would still appear to be that of (i) combining RCT and NRS without comment about the potential for NRS to bias the estimates, that is naively, or (ii) sub-setting by study type with or without reporting a pooled estimate, thus eliding the question of how best to deal with the inherent bias in NRS [7] and adopt a principled method of combining these different classes of information [8]. Failure to incorporate a principled analysis yields suspect inferential synthesis [9]. Albeit sub-setting RCT and NRS has been recommended [7, 10], the presentation of subgroupings and/or an overall estimate may result in reader extrapolation in a nontransparent manner based upon “…eyeballing…” the data and estimates [11]. The practical aspects refer to a lack of clarity with respect to appropriate search strategies for NRS within systematic reviews [12, 13].

The purpose of the current paper was first, to explore the soundness of estimating a pooled intervention effect [2] from meta-analyses combining RCT and NRS within a focused discipline, that of critical care [14–18]. A principled Bayesian method of combining information [8] via model averaging using the “bayesmeta” package [19, 20], as in previous studies [16, 21], was contrasted with conventional DerSimonian-Laird estimates (DSL [22]). A particular motivation was the suggestion, at least within the frequentist perspective, that the increase of sample size consequent upon the addition of NRS would increase effect estimate precision [4, 5]. Second, the utility of Bayes Factors, the posterior odds of one hypothesis when the prior probabilities of the two hypotheses under consideration are equal (BF [23]), was elucidated as a specific model selection criteria for either pooled or separate estimate(s) of RCT and / or NRS within meta-analyses. By way of such exploration the meta-analyses were fully characterized in the spirit of other studies [4, 5, 7, 24, 25]; that is, the paper conformed to a meta-research perspective [26]. By definition, the choice of meta-analyses addressing a diverse set of outcomes in the critically ill excluded a formal comparative effectiveness (CER) perspective (comparison of relative benefits and harms for a range of interventions for a given condition [12]), albeit such reviews may provide insight into the suitability of combining RCT and NRS within a single analysis.

## Methods

### Data acquisition

Published meta-analyses which combined RCT and NRS and reported a binary outcome, reflecting the importance of such outcomes in Critical-Care practice, were identified from the critical-care paradigm, using the electronic search engine Web of Science™. No attempt was undertaken to generate new meta-analyses by sourcing new individual RCT or NRS. The key words were: Meta-analysis / randomize controlled trials / observational studies /critically ill, or critical care, or intensive care; and specific journal searches: Intensive Care Medicine, Critical Care Medicine, Critical Care, Journal of Critical Care, Journal of Intensive Care Medicine, Chest, Thorax, Anesthesiology, Anaethesia, Annals of Surgery, Annals of Internal Medicine, JAMA, BMJ Open, PlosOne. Both adult and paediatric meta-analytic reports were included.

On the basis that, in the absence of strong informative priors, Bayesian analysis would be expected to generate wider parameter credible intervals than 95% frequentist confident intervals, the final meta-analytic cohort was chosen if the reported (frequentist) P-value of the pooled estimate (odds ratio (OR) or risk ratio (RR)) was < 0.05 and / or one of the study types (RCT or NRS) pooled estimate was < 0.05. All included non-RCT studies were classified, for analytic purposes, as NRS with the expectation that the number of RCT and non-RCT studies per meta-analysis would be small [27] and not susceptible to meaningful stratification.

### Statistical analysis

#### Bayesian approach

Although there are various methods to combine RCT and NRS [2, 6, 16], pooled meta-analytic estimates were established via the “bayesmeta” package (version 2.6 [19, 20]) within the R (version 4.3.1) statistical environment [28], as in previous studies [16, 21]; in particular, the R code in Appendix A.1 of Rover et al. [20]. Potential moderators of the pooled effects [18, 29] were not considered. This Bayesian approach was (i) based upon the normal-normal hierarchical model (NNHM) and (ii) used a two component model with an informative heavy-tailed mixture prior allowing for adaptive information sharing, whereby such sharing was stronger when RCT and NRS evidence were in agreement and weaker when they were in conflict [8, 20, 30]. That is, the Bayesian posterior constituted a model average, a weighted mixture of the conditional posteriors based upon the prior structures; specific data models corresponded to subgroupings (components) of the data with common or unrelated effects [20]. *It is in this sense that the notion of a principled approach to combining RCT and NRS is used.* The priors for the heterogeneity parameter ($\tau$) were half-normal and half-Cauchy [31] with scale 0.5 and a two component model was used [20]. The prior for the pooled effect estimate $\left( \mu \right)$ was normal, mean 0 and standard deviation 2, after Roever et al. [20]. Default credible intervals (CrI) of “bayesmeta” were computed as the shortest interval, which for unimodal posteriors (the usual case) was equivalent to the highest posterior density region [19]. Bayesian pooled estimates used the author metric (RR or OR).

Within the same Bayesian framework, model choice, in this case the preference for either a pooled estimate or separate estimates for both RCT and NRS, was addressed using Bayes Factors (BF [32, 33]). For probability model M fitted to data y, the marginal density of the data under model M is given as (we use the model syntax of Sinharay & Stern [34]):

$p\left( {y|{\text{M}}} \right) = \int {p\left( {y|\omega ,{\text{M}}} \right)} p\left( {\omega |{\text{M}}} \right)d\omega$, where $\omega$ is the parameter vector, the likelihood function is $p\left( {y|\omega ,{\text{M}}} \right)$ and the prior distribution for $\omega$ is $p\left( {\omega |{\text{M}}} \right)$. The BF for computing two models M_1_ and M_0_ is defined as:

${\text{BF}}^{10} = \frac{{p\left( {y|{\text{M}}_{1} } \right)}}{{p\left( {y|{\text{M}}_{0} } \right)}}$, the ratio of the marginal densities of the data ***y*** under the two models; thus the posterior odds equals BF x prior odds [34]. This being said, the determination of BF is a subject of some controversy [35]. BF were provided as part of the estimation routine (Appendix A.1 of [20]) for two-component models for half-normal and half-Cauchy heterogeneity priors. The utilised R code generated three “bayesmeta” objects: “bma.obs”, “bma.rct” and “bma.joint”. Marginal likelihoods were then computed as “pooled” (bma.joint_marginal) and “separate” (bma.obs_marginal*bma.rct_marginal) and Bayes Factors were subsequently derived for these marginal likelihoods as both “pooled” and “separate”; the latter being a reciprocal of the former. Model preference was accepted for BF_10_ > 3 or < 0.333 for the converse [33]. Posterior probabilities for the pooled estimate models were calculated, being derived from the posterior odds (posterior probability = posterior odds/(posterior odds + 1)), with model prior probabilities set to 0.5. Note the difference between (i) the within-model prior distribution(s) $p\left( {\theta |M_{i} } \right)$, the specification of the probability or uncertainty about the parameters within the model $M_{i}$ before observing the data and (ii) the model’s prior probability $p\left( {M_{i} } \right)$, the probability of the model holding as a whole; these two probabilities are independent. BF address the question of which model (strictly speaking, model class [36]) was more likely to have generated the data (y), whereas posterior model probabilities address the question of the plausibility of the model in light of the data $p\left( {M_{i} |y} \right)$ [37, 38].

### Frequentist approach

All meta-analytic frequentist pooled estimate were re-computed within Stata™ V17 [39] using the “metan” user-written module [40], current version 4.07 15th September 2023) with the DerSimonian & Laird random effects estimator (DSL [22]), as reflecting a conventional usage in meta-analytic statistical programs [16]. Variable distributions were compared with one-way analysis of variance and the effect of RCT proportion on the probability of both frequentist CI and Bayesian CrI excluding the null was estimated using logistic regression (robust variance) and marginal analysis (“margins command” [41]) within Stata™ V18. Frequentist statistical significance was ascribed at *P* < 0.05.

## Results

Fifty meta-analyses [42–91] were identified over calendar years 2009–2021. Twenty-nine were pharmaceutical-therapeutic and 21 were non-pharmaceutical therapeutic; author metric was OR in 23 and RR in 27. The median number of trials / studies, that is, RCT or NRS, was 9, minimum 2 and maximum 60, with 25th percentile 5 and 75th percentile 14. The median percentage of RCT was 0.33, minimum 0.05, maximum 0.80, with 25th percentile 0.20 and 75th percentile 0.57. Mortality was the most frequently reported outcome (50%), the other outcomes being various states consistent with the critically ill: clinical cure, intubation, acute kidney injury and venous thrombo-embolism. The most frequently used statistical programs were RevMan (https://training.cochrane.org/online-learning/core-software-cochrane-reviews/revman, 62%), Stata™ (https://www.stata.com/, 16%), Comprehensive Meta-Analysis (https://www.meta-analysis.com/, 6%) and R (https://www.r-project.org/, 6%). All meta-analyses used a primary frequentist method of analysis: DerSimonian-Laird (DSL) random effects (RE) in 14; Mantel–Haenszel RE (M-H RE) in 18; M-H fixed effects (M-H FE) in 4 (with I^2^ values of 27, 32, 43 and 49%); RE not specified in 8; and model not specified in 6. Heterogeneity was also varyingly reported as $\tau^{2}$ and / or I^2^. Of the 4 studies using M-H FE estimation this decision was made on the criterium of heterogeneity (I^2^ < 50%) without further justification. Similar reasons (I^2^ > 50%) for choosing a RE approach were also given as was the disparateness of individual RCT / NRS within a meta-analysis. Of note, no meta-analysis discussed the impact of small study number in meta-analyses [16] or utilized alternate frequentist variance estimators such as the the Hartung-Knapp-Sidik-Jonkman (HKSJ) method [16] for adjusting tests and intervals as recommended by Bender et al. [92] in small RCT number meta-analyses. Only one meta-analysis used a Bayesian method in a sensitivity analysis to test the “robustness” of frequentist results [53].

Author reasons [25] for combining RCT and NRS varied considerably: a brief statement that such would be done, the wish to use all or the best available evidence [93] and the small number of RCTs addressing the meta-analytic question(s) of interest. Three meta-analyses did not detail quality assessment: in Chiumello et al. [48], the latter was not mentioned; in Tagaki et al. [76], adjusted NRS studies were provided; and in Wan et al. [81], as the NRS were not the primary focus, albeit both adjusted and non-adjusted NRS estimates were given. The Cochrane Collaboration risk of bias tool for RCT was the most frequently used [94], also the Jadad score [95] and the RoB2 instrument [96]; for NRS the Newcastle–Ottawa Scale [97] predominated, as well as the Robins-I [98] and MINORS [98] instruments.

An overall pooled estimate produced by combining RCT and NRS was reported in 42 meta-analyses considered. With respect to study type, reported P-values for effect estimates were < 0.05 in 11/26 (42%) statistical analyses for RCT, 18/24 (75%) for NRS, and 37/42 (88%) for pooled estimates (RCT and NRS) within a single meta-analysis report. Pooled recomputed frequentist DSL estimates were significant in 78% (39/50); 36% (18/50) in RCT and 60% (30/50) in NRS.

**Fig. 1:**
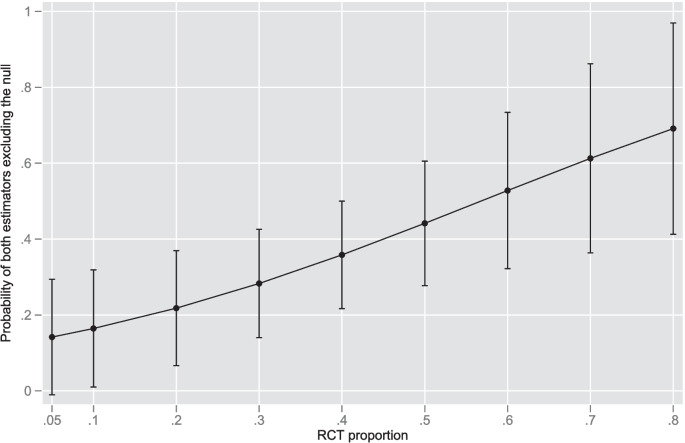
Probability (with 95% CI) of both frequentist CI and Bayesian CrI excluding the null (within the same meta-analysis) as a function of RCT proportion

For Bayesian estimation using the half-normal heterogeneity prior (*n* = 49), significant effects (CrI excluding the null) were observed in 18/4 (37%); for the half-Cauchy prior (*n* = 49), 15/49 (31%). For the meta-analytic reports where Bayesian CrI could be computed (see below), pooled (RCT and NRS) estimates demonstrated the following (within the same meta-analytic report): in eighteen meta-analytic reports (37.5%), seven in the OR and eleven in the RR metric, there was agreement between frequentist CI and Bayesian CrI in achieving statistical significance; in twenty-three (48%) meta-analysis reports where frequentist pooled CI achieved statistical significance, Bayesian CrI did not achieved statistical significance; in seven meta-analyses both frequentist CI and Bayesian pooled CrI did not achieved statistical significance. Of interest, the RCT proportion, not the number of studies (both RCT and NRS) appeared determinant with respect to the probability of both frequentist CI and Bayesian CrI in excluding the null (within the same meta-analysis), as seen in Fig. 1.

Two Bayesian estimates were computed, corresponding to the half-normal and half-Cauchy heterogeneity priors. For one meta-analysis, Barakakis et al. [44], two RCT and one NRS, no Bayesian CrI could be computed. For the Sultan et al. meta-analysis [74], one RCT and one NRS, Bayesian CrI could only be computed for the half-Cauchy heterogeneity prior models, and for Wang et al. [83], three NRS and one RCT, Bayesian CrI could only be computed for the half-normal heterogeneity prior model. The study of Yao et al. [86] presented results in the risk difference metric (RD), 0.099( 0.015, 0.184); as all other estimation results were in the OR or RR metric, RR was utilised.

Table 1 lists the author and Bayesian estimates of the two-component models for half-normal and half-Cauchy heterogeneity priors respectively. All Bayesian estimates for meta-analyses having an author overall-estimate *P*-value > 0.05 were consistent in terms of the span of CrIs, that is, they encompassed unity.

**Table 1:** Author and Bayesian (half-normal heterogeneity prior) estimates

Author	Ref	Author	Author	Author	Author	BM2_N	BM_N	BM_N	BM_C	BM_C	BM_C
		metric	estimate	lcl	ucl	estimate	lcl	ucl	estimate	lcl	ucl
Akingboye	42	OR	0.55	0.33	0.9	0.563			0.629		
Aoyama	43	RR	1.44	1.11	1.88	1.414	0.8	2.361	1.418	0.712	2.605
Barakakis	44	OR	0.78	0.64	0.96						
Beks	45	RR	0.41	0.27	0.61	0.446	0.226	1.604	0.442	0.226	1.544
Bellos	46	OR	0.34	0.16	0.69	0.414	0.158	1.127	0.419	0.128	1.568
Chan	47	RR	1.33	1.22	1.46	1.297	1.036	1.659	1.295	1.042	1.641
Chiumello	48	RR	0.26	0.09	0.71	0.306	0.102	0.937	0.307	0.098	0.987
Cortegiani	49	OR	0.74	0.55	0.98	0.721	0.352	1.352	0.72	0.3	1.564
De Jong	50	OR	2.07	1.35	3.16	1.72	0.908	3.094	1.718	0.869	3.34
Ding	51	OR	0.39	0.3	0.51	0.338	0.224	0.515	0.339	0.213	0.548
Eom	52	OR	1.257	0.972	1.625	1.261	0.974	1.647	1.26	0.972	1.647
Fiolet	53	RR	1.27	1.04	1.54	1.091	0.305	1.741	1.102	0.253	1.956
Flannery	54	OR	0.47	0.34	0.65	0.649	0.338	1.317	0.648	0.331	1.322
Hammond	55	RR	0.86	0.75	0.99	0.921	0.692	1.155	0.915	0.689	1.143
Kherad	56	OR	0.86	0.72	1.02	0.937	0.31	2.618	0.938	0.312	2.741
Lee	57	RR	0.838	0.691	1.016	0.862	0.581	1.344	0.855	0.576	1.319
Leinicke	58	RR	0.44	0.28	0.69	0.425	0.24	1.657	0.422	0.222	1.919
Liu	59	RR	1.44	1.17	1.76	1.407	1.096	2.05	1.403	1.1	2.036
Luo	60	RR	0.77	0.66	0.89	0.686	0.528	0.89	0.686	0.531	0.882
Mao	61	OR	0.44	0.23	0.83	0.542	0.255	1.123	0.532	0.225	1.209
Mei	62	OR	3.06	1.15	8.15	5.178	1.138	36.078	4.364	0.978	39.344
Poirier	63	OR	0.711	0.393	1.288	0.446	0.226	1.604	0.442	0.226	1.544
Price	64	OR	1.25	1.06	1.48	1.076	0.688	2.041	1.075	0.654	2.162
Ramesh	65	RR	0.63	0.42	0.93	0.623	0.341	1.123	0.624	0.334	1.556
Ribeiro	66	RR	0.49	0.35	0.69	0.609	0.147	2.13	0.611	0.143	2.26
Schneider	67	RR	1.73	1.35	2.2	1.457	0.786	2.211	1.525	0.804	2.23
Shao	68	OR	0.463	0.332	0.646	0.63	0.352	1.142	0.592	0.346	1.127
Shen	69	RR	0.85	0.73	0.98	0.792	0.659	0.97	0.791	0.663	0.96
Shim	70	RR	0.72	0.59	0.9	0.717	0.427	0.99	0.721	0.417	1.006
Silva	71	OR	0.66	0.52	0.84	0.662	0.461	0.953	0.662	0.467	0.942
Sklar	72	RR	0.81	0.67	0.96	0.821	0.431	2.915	0.819	0.393	3.21
Stephens	73	OR	0.34	0.21	0.54	0.56	0.216	2.746	0.495	0.205	2.913
Sultan	74	RR	2.92	0.481	17.741	1.831			1.418	0.718	2.605
Sun	75	OR	0.43	0.32	0.58	0.494	0.269	0.917	0.493	0.269	0.917
Tagaki	76	OR	0.423	0.331	0.539	0.847	0.365	1.275	0.836	0.357	1.265
Tang	77	RR	0.62	0.43	0.91	0.65	0.362	0.965	0.654	0.349	0.976
Teo	78	RR	0.66	0.53	0.83	0.689	0.507	0.955	0.687	0.511	0.933
Tlayeh	79	RR	0.649	0.488	0.864	0.704	0.46	1.127	0.7	0.453	1.134
Tsaousi	80	RR	0.634	0.496	0.809	0.644	0.406	1.094	0.642	0.393	1.333
Wan	81	RR	0.598	0.529	0.675	0.643	0.5	1.214	0.635	0.499	1.174
Wang	82	OR	1.43	1.3	1.59	0.757	0.459	1.431	0.778	0.455	1.448
Wang	83	RR	0.353	0.183	0.68	0.297	0.058	0.753	0.315		
Wieczorek	84	OR	1.22	1	1.5	1.224	0.864	1.78	1.222	0.866	1.757
Yang	85	OR	1.88	1.29	2.73	1.842	1.059	3.206	1.843	1.043	3.266
Yao	86	RR	1.933	1	3.737	1.204	0.445	3.625	1.224	0.359	4.572
Ye	87	OR	0.25	0.13	0.48	0.27	0.1	0.71	0.273	0.089	0.862
Yedlapati	88	RR	0.82	0.8	0.84	0.433	0.276	0.899	0.439	0.273	0.948
Yu	89	OR	2.1	1.31	3.38	1.98	1.185	3.261	1.988	1.171	3.333
Zakhari	90	RR	0.41	0.26	0.65	0.466	0.314	0.654	0.472	0.322	0.657
Zampieri	91	RR	1.23	1.05	1.43	1.208	0.974	1.476	1.21	0.99	1.469

Author estimate / lcl / ucl: derived from Der-Simonia Laird re-estimation of the pooled estimate of RCT & NRS (see Statistical analysis Frequentist Approach). Ref. Reference. B_N, Bayesian model with half-normal heterogeneity prior. B_C, Bayesian model with half-Cauchy heterogeneity prior. lcl, lower 95%CI. ucl, upper 95%CI. lcrl, lower 95% credible interval

**Fig. 2:**
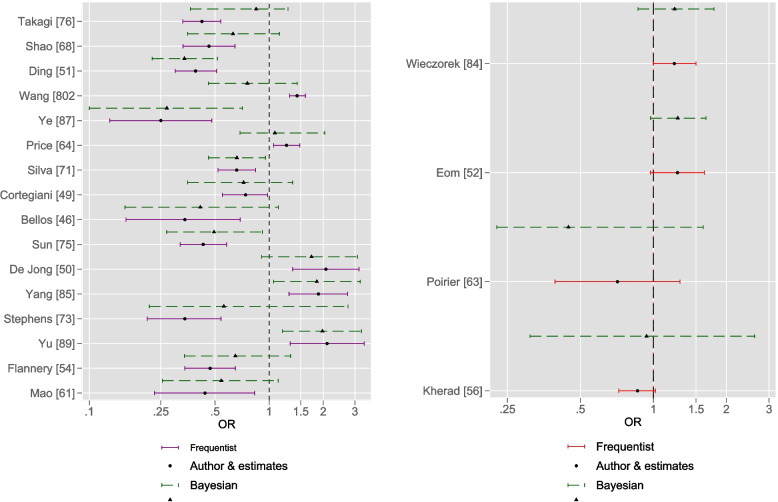
Author (frequentist) and Bayesian estimates as couplets for OR metric, with X-axis on the log scale. Significant (left panel) and nonsignificant (right panel) overall OR frequentist estimates compared with Bayesian estimates (half-normal heterogeneity parameter ($\tau$)). Due to scaling requirements, estimates from the Mei et al. meta-analysis [62] were omitted (frequentist estimate: 3.06(1.15, 8.15); Bayesian: 5.18(1.14, 36.08))

**Fig. 3:**
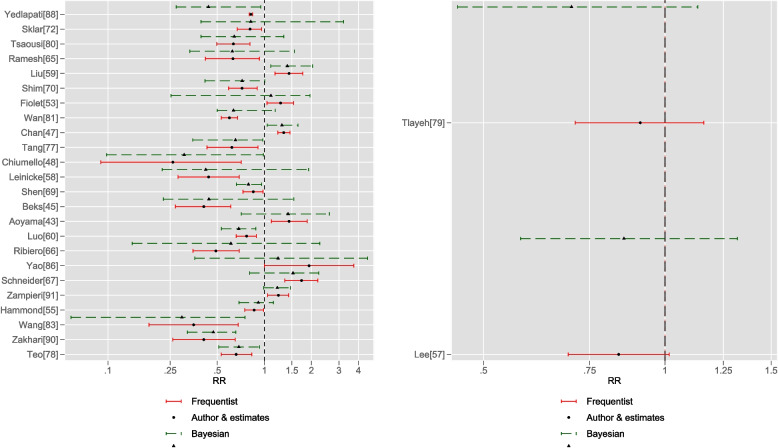
Author (frequentist) and Bayesian estimates as couplets for RR metric, with X-axis on the log scale. Significant (left panel) and nonsignificant (right panel) overall RR frequentist estimates compared with Bayesian estimates (half-Cauchy heterogeneity parameter ($\tau$)). Due to scaling, estimates from the Sultan et al. meta-analysis [74] were omitted (frequentist estimate: 2.92(0.481, 17.741); Bayesian: 1.418(.718, 2.605))

A graphical comparison of the author (frequentist) and Bayesian estimates as couplets for OR (Fig. 2) and RR (Fig. 3) was undertaken to further illustrate these differences. With regard to Fig. 2, in six of the meta-analyses, both frequentist CI width and corresponding Bayesian CrI width excluded the null; all Bayesian CrI spans were greater than frequentist CI spans.

With regard to Fig. 3, in nine of the meta-analyses, both frequentist CI width and corresponding Bayesian CrI width excluded the null; in two meta-analyses, author frequentist CI width was greater than Bayesian CrI width: Zakhari et al. [90]: RR 0.41(0.26, 0.65) versus 0.472(0.322, 0.657) and Sultan et al. [74]: RR 2.92(0.481, 17.741) versus 1.418(0.718, 2.605).

Preference for either a pooled or separate estimates within the two-component models using BF criteria is shown in Table 2. Note that the descriptor “Separate” in the legend to Table 2 refers to the generation of BF from a single marginal likelihood (dervide from the multiplication of bma.obs_marginal*bma.rct_marginal: see Statistical analysis Bayesian approach, above).

**Table 2:** BF and posterior model probabilities for model preference for half-normal and half-Cauchy heterogeneity priors. BF > 3, bolded

Half-normal					Half-Cauchy						
Author	Ref	Pooled	Separate	PPp	Study number	RCT	NRS	Metric	Pooled	Separate	PPp
Akingboye	42	1.541	0.649	0.606	4	1	3	OR	1.626	0.615	0.619
Aoyama	43	**4.350**	0.230	0.813	4	1	3	RR	**4.743**	0.211	0.826
Barakakis	44										
Beks	45	2.567	0.390	0.720	25	3	22	RR	**3.158**	0.317	0.759
Bellos	46	2.177	0.459	0.685	3	1	2	RR	2.231	0.448	0.690
Chan	47	**8.176**	0.122	0.891	7	3	4	RR	**10.957**	0.091	0.916
Chiumello	48	1.693	0.591	0.629	5	4	1	RR	1.766	0.566	0.638
Cortegiani	49	**3.301**	0.303	0.768	4	1	3	OR	**3.574**	0.280	0.781
De Jong	50	**6.564**	0.152	0.868	9	3	6	OR	**6.548**	0.153	0.868
Ding	51	**9.136**	0.109	0.901	13	2	11	OR	**8.788**	0.114	0.898
Eom	52	2.703	0.370	0.730	28	23	5	OR	**3.032**	0.330	0.752
Fiolet	53	1.842	0.543	0.648	7	1	6	RR	2.060	0.485	0.673
Flannery	54	**3.437**	0.291	0.775	11	2	9	OR	**4.096**	0.244	0.804
Hammond	55	0.698	1.433	0.411	10	6	4	RR	0.876	1.141	0.467
Kherad	56	2.441	0.410	0.709	9	4	5	OR	2.812	0.356	0.738
Lee	57	2.725	0.367	0.732	9	2	7	RR	**3.512**	0.285	0.778
Leinicke	58	2.357	0.424	0.702	5	1	4	RR	2.628	0.381	0.724
Liu	59	**6.936**	0.144	0.874	6	4	2	RR	**8.830**	0.113	0.898
Luo	60	**4.229**	0.236	0.809	32	23	9	RR	**5.700**	0.175	0.851
Mao	61	1.142	0.875	0.533	5	2	3	OR	1.336	0.749	0.572
Mei	62	0.543	1.842	0.352	6	2	4	OR	0.709	1.411	0.415
Poirier	63	0.014	**71.502**	0.014	23	8	15	OR	0.023	**44.149**	0.022
Price	64	**4.781**	0.209	0.827	19	1	18	OR	**5.385**	0.186	0.843
Ramesh	65	**4.017**	0.249	0.801	7	3	4	RR	**4.773**	0.210	0.827
Ribeiro	66	2.107	0.475	0.678	7	2	5	RR	2.359	0.424	0.702
Schneider	67	0.903	1.108	0.474	23	7	16	RR	1.189	0.841	0.543
Shao	68	0.694	1.442	0.410	24	8	16	OR	0.914	1.094	0.478
Shen	69	**9.118**	0.110	0.901	18	2	16	RR	**11.863**	0.084	0.922
Shim	70	**4.340**	0.230	0.813	4	3	1	RR	**4.772**	0.210	0.827
Silva	71	**7.190**	0.139	0.878	8	4	4	OR	**9.473**	0.106	0.905
Sklar	72	2.701	0.370	0.730	5	1	7	RR	**3.029**	0.330	0.752
Stephens	73	0.690	1.449	0.408	9	2	7	OR	0.843	1.186	0.457
Sultan	74	1.018	0.982	0.504	2	1	1	RR	1.034	0.967	0.508
Sun	75	**3.721**	0.269	0.788	14	3	11	OR	**4.559**	0.219	0.820
Tagaki	76	0.183	**5.473**	0.154	26	4	22	OR	0.243	**4.115**	0.195
Tang	77	**5.104**	0.196	0.836	9	4	5	RR	**5.981**	0.167	0.857
Teo	78	**6.388**	0.157	0.865	21	12	9	RR	**8.782**	0.114	0.898
Tlayeh	79	2.926	0.342	0.745	13	3	10	RR	**3.635**	0.275	0.784
Tsaousi	80	**5.042**	0.198	0.834	4	3	1	RR	**5.453**	0.183	0.845
Wan	81	2.077	0.482	0.675	27	4	23	RR	2.759	0.362	0.734
Wang	82	1.111	0.900	0.526	4	1	3	OR	1.203	0.831	0.546
Wang	83	0.204	**4.914**	0.169	60	9	51	OR	0.270	**3.704**	0.213
Wieczorek	84	**5.707**	0.175	0.717	10	2	8	OR	**7.367**	0.136	0.880
Yang	85	**3.758**	0.266	0.790	9	5	4	OR	**4.217**	0.237	0.808
Yao	86	0.648	1.544	0.393	6	2	4	RR	0.769	1.300	0.435
Ye	87	**3.026**	0.330	0.752	5	1	4	OR	**3.188**	0.314	0.761
Yedlapati	88	0.257	**3.895**	0.204	7	4	3	RR	0.312	**3.202**	0.238
Yu	89	2.889	0.346	0.743	10	6	4	OR	**3.469**	0.288	0.776
Zakhari	90	0.524	1.908	0.344	14	11	3	RR	0.323	**3.098**	0.244
Zampieri	91	**7.673**	0.130	0.885	11	5	6	RR	**10.207**	0.098	0.911

*Half-normal* Half-normal heterogeneity prior, *Half-Cauchy* Half-Cauchy heterogeneity prior, *Ref*. Reference. Pooled, BF for pooled estimates (RCT & NRS). Separate, BF for “separate” estimates of RCT & NRS (ie, derivation from marginal likelihoods as: bma.obs_marginal*bma.rct_marginal). “Pooled” and “Separate” estimation of BF were derived from the appropriate marginal likelihoods. The “Separate” BF values were the reciprocal of the”Pooled” (see: “Statistical analysis, Bayesian, above). *PPp *Posterior model probability

For the half-normal heterogeneity prior, 21 meta-analyses favored pooling (RR, 11 and OR, 10) and 4 favored separate analysis (RR, 1 and OR,3) with BF > 3. For the half-Cauchy heterogeneity prior, 27 meta-analyses favored pooling (RR, 15 and OR, 12) and 4 favored separate analysis (RR, 2 and OR,2) with BF > 3. Analysis of the table information did not yield convincing predictors of BF > 3 with respect to metric or meta-analytic study number(s).

## Discussion

The current study demonstrated a substantial reduction in the nominal frequentist significance of meta-analytic estimates generated by the naïve pooling of RCT and NRS (using the DSL estimator) compared with a principled Bayesian method of information combination. The latter, a model averaging process, adjusted for the agreement or otherwise between the RCT and NRS studies offsetting the increase in frequency of statistically significant (frequentist) treatment effects of NRS studies compared with RCT, within the same meta-analysis report. A plausible expectation that a Bayesian approach would yield a frequency of statistically significant (CrI excluding the null) pooled meta-analyses comparable with that of significant RCTs within a frequentist DSL analysis was also realized: Bayesian 37% (half-normal heterogeneity prior) and 31% (half-Cauchy) compared with DSL 36%.

Several studies have addressed potential conflict between RCT and NRS effect estimate combination with various purposes and results: an endorsement of such combination [25], a finding of consistent direction of overall effect [24] or little difference between the effect estimates [7, 99, 100], and the promise of increased precision of effect consequent upon larger sample size [4, 5]. The analytic assumption behind these studies was frequentist. A larger CI span has also been suggested [10, 101] but, as noted above, a precision increase was not generally found in the current study, more so with the application of Bayesian methods.

Proposals to incorporate randomized and non-randomized evidence within meta-analyses have a considerable history of at least 30 years [102], as has the particular question of the bias or otherwise of NRS [103, 104]. The methodological issues involved in such exercises have been considered in some detail [10, 101, 105, 106]. A general statistical framework to combine multiple information sources was first introduced in 1989 [6, 16], the Confidence Profile Method, and the recent (2021) paper by Nikolaidis et al. provides a more current review of information sharing categories ([8], Fig. 3) as: functional, deterministic functions relating to model parameters of both direct and indirect evidence; exchangeability, a common distribution imposed upon a parameter set; prior-based, a Bayesian method utilizing an informative prior to combine evidence, to wit, the “bayesmeta” approach [20]; and multivariate, whereby a multivariate distribution is imposed across parameters specifying outcomes, not populations or study designs [15]. A plethora of Bayesian models have been proposed to combine direct and indirect evidence and have been usefully summarized in a number of papers [2, 6, 107–109] and briefly detailed [16]; this theme is not pursued here.

The “bayesmeta” approach [20] seemed ideally suited to the task at hand; available through the R computing environment and syntax: a computationally efficient method, using numerical integration and analytical tools, not Markov Chain Monte Carlo, with heavy-tailed priors for effect estimation resulting in a model-averaging technique. This approach has been pursued in recent studies [110, 111]. The described method was robust [30] in the sense that a potential prior-data conflict, that is, a discrepancy between source and target data, was explicitly projected. The “bayesmeta” program formulates a random effects normal-normal hierarchical model [19, 20] and there has been some discussion, albeit indeterminate, regarding the impact of the normality assumption [112–114]. The experience of Davey et al. that the median number of studies per review in the Cochrane Database of Systematic Reviews was six (inter-quartile range (IQR) 3–12) was consistent with that of the current study (median 9, IQR 5–14). No marked effect of the heterogeneity prior was evident in that point and CrI estimates of the different models, half- normal and Cauchy heterogeneity priors, were comparable and convergence difficulties [115] were not a major issue although (see Results, Tables 1 and 2) no CrI could be computed in two meta-analyses and selective computation occurred for either half-normal or half-Cauchy priors in two.

**Fig. 4:**
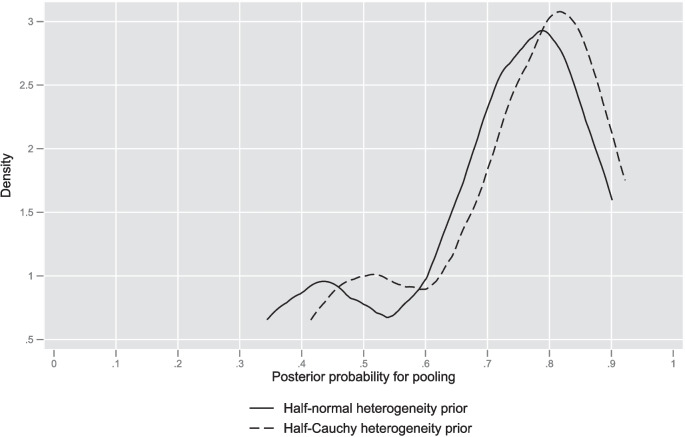
Kernel density plots of posterior model probabilities of pooling for meta-analyses where 0.333 < BF < 1

Preference for the pooled analysis (RCT plus NRS) via BF was indicated in 42% and 54% of meta-analyses depending upon the heterogeneity prior (Table 2). BF are known to be sensitive to model parameter prior distribution, and the fact that different priors result in different BF should “… not come as a surprise” [116]. A kernel density plot (Fig. 4) of the posterior probabilities for the pooled model for both heterogeneity priors, where BF for model choice were indeterminant (0.333 < BF < 1), revealed the highest posterior densities located close to 0.8, giving further support to the pooled model formulation for this subgroup of meta-analyses.

### Limitations

Different approaches to information combination were not explored, as in a previous study, where, with respect to a single exemplar meta-analysis combining RCT and NRS, non-naïve methods, both frequentist and Bayesian, were consistently shown to generate CI and CrI widths embracing the null, as opposed to the simple DSL estimator ([16], Table 2, page 53). It was instructive to note that none of the currently considered meta-analyses reported using non-DSL estimators, despite concerns being raised nearly 10 years ago about biased estimates with falsely high precision with DSL estimator [117]. As a reviewer pointed out, such an observation goes to the heart of the difference between the handling of heterogeneity between the two paradigms: frequentist, where the heterogeneity variance (τ^2^) is a fixed quantity, albeit it may vary with different frequentist estimators ([118] and see below) and Bayesian, where prior distributions are specified for the heterogeneity parameter [119]; in the current study, half-normal and half-Cauchy. As noted by Rover et al., within Bayesian estimation the choice of a prior for $\tau^{2}$ is a somewhat nuanced process [120]. Such considerations have been further explored by Rover et al., including the effect of the scaling of the prior whereby the latter was found to have more impact upon results than the prior distribution shape [121]; the current study used a scale of 0.5 for both heterogeneity priors. Rover et al. [120] also found that mortality endpoints in a cohort of meta-analyses from the Cochrane Database of Systematic Reviews had a comparatively low heterogeneity compared with other outcomes. A similar review, by Inthout et al. [122], found that meta-analyses with a dichotomous outcome had τ values (the square-root of $\tau^{2}$ and on the same scale as the effect size metric) of 0(0–0.41); median, interquartile (Q1-Q3)). If we consider values of $\tau$ in the range of 0.1–0.5 as reflecting small to moderate heterogeneity [123], then the half-Cauchy distribution would ensure that a value less than τ = 0.4 has a probability of 43% and for the half-normal distribution, 58%; suggesting weakly informative priors for such a scenario ([119], computations performed in the R package “extraDistr” version 1.10.0; @ https://cran.r-project.org/web/packages/extraDistr/index.html). For comparison with the current study, the overall $\tau$(median, interquartile (Q1-Q3)) for the combined estimate of RCT and NRS (50 meta-analyses) using the DSL estimator (see Supplement, Table S1) was 0.25(0.10–0.50).

These observations have relevance to the present study with respect to the “disagreements” between the DSL CI and the Bayesian model averaging CrI with respect to the null. A large number of frequentist meta-analytic estimators are provided by the Stata “metan” user-written module [124] and some of these were used in the original published meta-analyses. The Mantel–Haenszel RE (M-H RE) estimator would appear to be available only in “RevMan” software, but with respect to any differences between the DSL and M-H RE estimators, the Cochrane Handbook "Implementing random-effect mete-analyses" (10.4.4) [125], notes that the difference between the DSL and M-H random effects approaches would be "likely to be trivial". The question of the appropriate estimator choice, fixed or random, is not canvassed in this paper; suffice it to say, the (qualified) comment of Borenstein et al. is noted: “in the vast majority of meta-analyses the random-effects model would be the more appropriate choice” [126].

As suggested by a reviewer, two alternate frequentist meta-analytic estimators were also compared with the Bayesian model in terms of the “disagreements”, as above: (i) the Hartung-Knapp-Sidik-Jonkman (HSJK) variance correction (to any standard tau-squared estimator, in this case, the DSL estimator) [127–129] and (ii) the inverse-variance heterogeneity model (IVhet) of Doi and colleagues [130, 131]. As these comparisons were not the prime focus of the current paper, they are only summarized here and presented in detail for the reader in the Supplement.

For the HJKS variance correction with the DSL estimator (HJKS-DSL), 55% (27/49, no HSJK-DSL estimates could be computed for the Sultan et meta-analysis [74]) were significant compared with 78% using the conventional DSL estimator. In the OR metric for significant HJKS-DSL estimates (CI not spanning the null), 4 Bayesian CrI spanned the null. For non-significant HJKS-DSL estimates (CI spanning the null), all Bayesian estimates were consistent (Figure S1). In the RR metric (Figure S2), for significant HJKS-DSL estimates (CI not spanning the null), 9 Bayesian CrI spanned the null. For non-significant HJKS-DSL estimates (CI spanning the null), 2 Bayesian estimates did not span the null.

For the Doi et al. IVhet model, 58% (29/50) were significant compared with 78% using the conventional DSL estimator. In the OR metric (Figure S3) for significant IVhet estimates (CI not spanning the null), 6 Bayesian CrI spanned the null. For non-significant IVhet estimates (CI spanning the null), all Bayesian estimates were consistent. In the RR metric (Figure S4) for significant IVhet estimates (CI not spanning the null), 9 Bayesian CrI spanned the null. For non-significant IVhet estimates (CI spanning the null), 1 Bayesian estimate did not span the null.

#### Future possibilities

In 2009 Sutton et al. [132] suggested that evidence synthesis was the “the key to more coherent and efficient research” and posed the question whether “evidence from observational studies may exist which could augment that available from the RCTs”. A decade on, the answer would appear to be affirmative, at least from a Bayesian perspective. Any combination of RCT and NRS is predicated upon preceding robust study quality assessment; for instance, a checklist that may be applied to both RCT and NRS, such as that of Downs and Black [133] used by Sampath et al. [18]. The former was described as being “suitable for use in a systematic review” [104]. The question of combining RCT and NRS under conditions of “conflict” between conclusions can only be achieved by a principled approach, such as Bayesian model averaging as described above, complemented by BF computation. This being said, the umbrella term NRS, as used in the current study, elides a potential number of important (non-randomised) study types, such as prospective and retrospective, cross-sectional and longitudinal, observational and interventional.

Future studies should replicate or otherwise the findings of the current study, including the utility of BF, model posterior probabilities and different non-randomised study designs. In any concurrent comparison with frequentist estimator(s), the latter choice should be justified; such comparisons are presented for the reader in the online Supplement.

## Conclusions

Bayesian estimation of treatment efficacy via model averaging was more conservative than frequentist in meta-analyses combining NRS and RCT. The calculation of BF was able to provide additional evidence for the wisdom or otherwise of meta-analytic pooling of RCT and NRS. Model posterior probabilities also provided plausible evidence for the pooled estimate model. If frequentist estimators are utilized, caution should attend estimator choice and the reporting of meta-analytic pooled estimates.

### Supplementary Information

**Supplementary Material 1.**

## Data Availability

The data sets used for the paper are under the proprietorship of the authors (JLM and AL) and can be acquired from the corresponding author (JLM) upon reasonable request.
