# Supplementary material for: Problematic meta-analyses: Bayesian and frequentist perspectives on combining randomized controlled trials and non-randomized studies
Source: BMC Med Res Methodol. 2024 Apr 27;24:99. doi: 10.1186/s12874-024-02215-4 (PMC11056075; doi:10.1186/s12874-024-02215-4)
Supplement: Supplementary file 1 — Supplementary Material 1. [file 12874_2024_2215_MOESM1_ESM.docx]

**Supplement**

The purpose of the supplement is to explore the performance of two alternate frequentist meta-analytic estimators (i) the Hartung-Knapp-Sidik-Jonkman (HSJK) variance correction (to any standard tau-squared estimator, in this case, the DSL estimator) [1-3] and (ii) the inverse-variance heterogeneity model (IVhet) of Doi and colleagues [4, 5] with respect to the Bayesian model averaging estimator implemented in the main paper. Details of the heterogeneity estimates of the meta-analytic cohort are also provided in Table S1.

**Hartung-Knapp-Sidik-Jonkman (HSJK) correction**

Compared with the DSL estimator, the HSJK correction produced far more conservative estimates with respect to the null; pooled frequentist estimates were significant in 55% (27/49, HSK-DSL) versus 88% (44/50, DSL) versus; no HSJK estimates could be computed for the Sultan et meta-analysis [6]).

Of interest, although producing more adequate error rates than the DSL method, the HSJK correction has not been recommended (“extra caution is needed”) with meta-analyses of “≤ 5 studies of very unequal sizes” [1]. In the current paper there were 13 meta-analyses where study number was ≤ 5 and the patient numbers per study (RCT plus NRS) ranged from 182-6908. The HSJK correction is typically untruncated and if its value is <1 the confidence interval around the pooled effect may be less conservative than under a model without the HKSJ correction [2]. Under these circumstances the Stata module “metan” [7] issues a warning with a recommendation to truncate the correction to one. This “truncate” option was applied to analysis in 5 [8-12] meta-analyses. Only one meta-analysis, Chiumello [8], had a low (5) study number. As in the main paper, two graphs for the HJKS-DSL estimator are presented in the OR and RR metrics respectively.

In the OR metric (Figure S1), for significant HSK-DSL estimates (CI not spanning the null), 4 Bayesian CrI spanned the null. For non-significant DSL estimates (CI spanning the null), all Bayesian estimates were consistent.

Figure S1. Author (frequentist: HJKS-DSL) and Bayesian estimates as couplets for OR metric with X-axis on the log scale. Significant (left panel) and nonsignificant (right panel) overall OR frequentist estimates compared with Bayesian estimates (half-normal heterogeneity parameter ($\tau$)). Due to scaling requirements, estimates from the Mei et al meta-analysis [13] were omitted (frequentist estimate: 3.04(0.865, 10.68); Bayesian: 5.18(1.14, 36.08)).


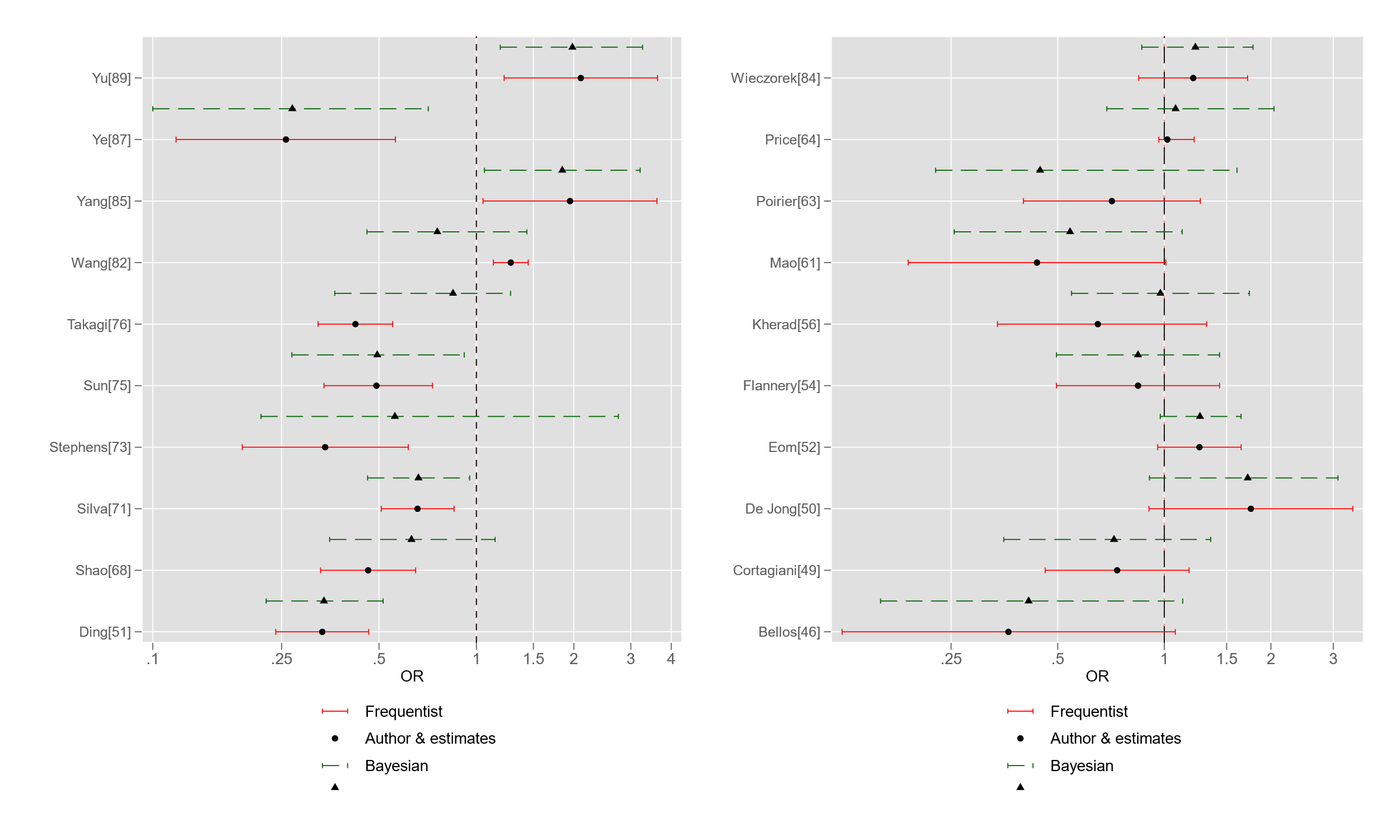


In the RR metric (Figure S2), for significant DSL estimates (CI not spanning the null), 9 Bayesian CrI spanned the null. For non-significant DSL estimates (CI spanning the null), 2 Bayesian estimates did not span the null.

Figure S2. Author (frequentist: HJKS-DSL) and Bayesian estimates as couplets for RR metric with X-axis on the log scale. Significant (left panel) and nonsignificant (right panel) overall RR frequentist estimates compared with Bayesian estimates (half-Cauchy heterogeneity parameter ($\tau$)). No HJKS estimates were available for the Sultan et al meta-analysis [6]).

**
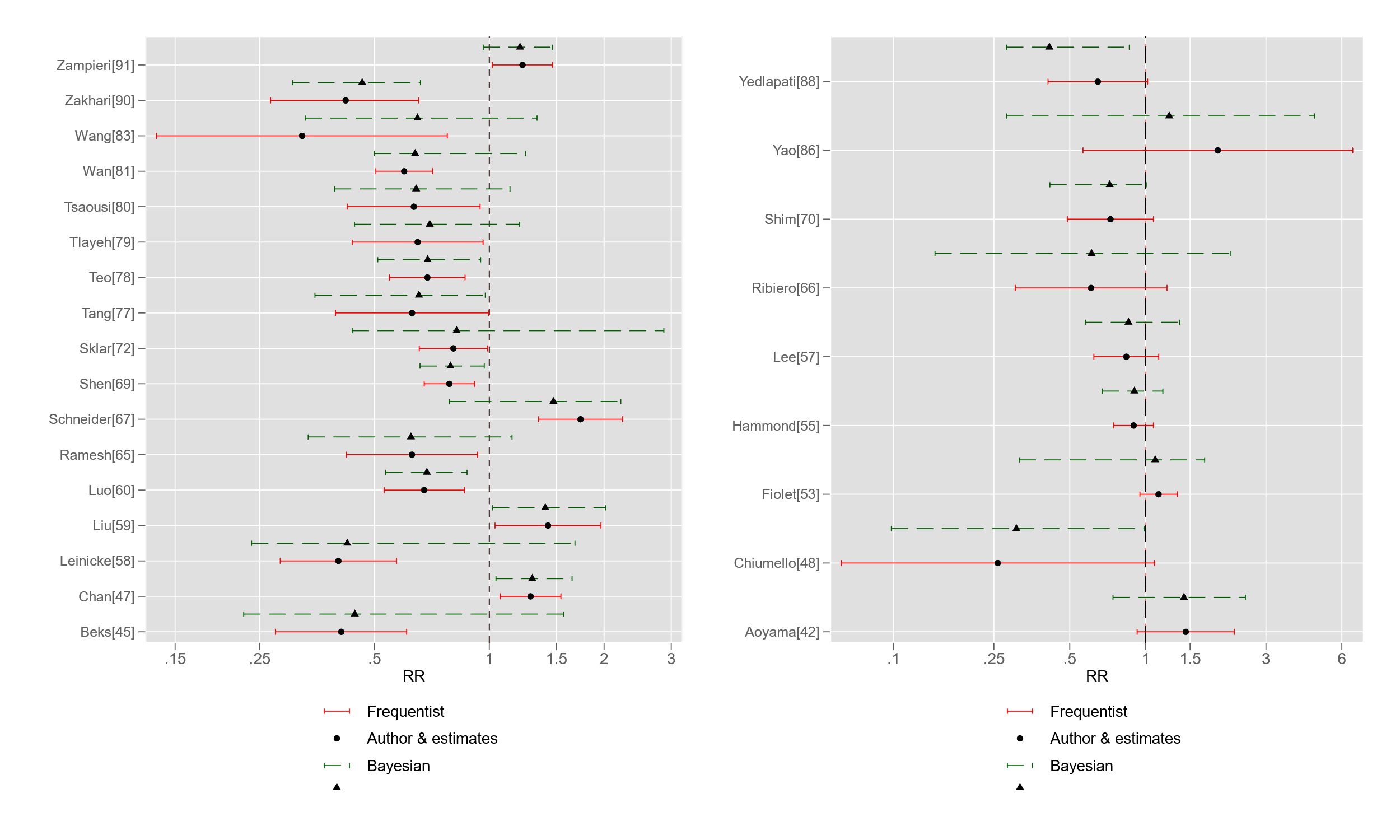
**

**Inverse-variance heterogeneity (IVhet) model of Doi and colleagues**

Summarizing the exposition in Doi et al [5]: the IVhet estimator produces point estimates under the fixed effect (FE) model assumptions (IVhet and FE point estimates are identical) with a quasi-likelihood based variance structure. For the FE estimator, as heterogeneity increases, there is variance overdispersion, that is, greater variance than theoretically expected. For the random effects (RE) estimator, individual study treatment effects differ and follow a normal distribution and there is a common variance which tends to redistribute variance from larger to smaller studies. In the presence of low heterogeneity, variances in the RE and IV het models are similar and diverge with increase in heterogeneity.

Compared with the DSL estimator, the IVhet estimator again produced far more conservative estimates with respect to the null; IVhet estimates were significant in 58% (29/50) versus 88% (44/50, DSL).

Figure S3. Author (frequentist: IVhet) and Bayesian estimates as couplets for OR metric with X-axis on the log scale. Significant (left panel) and nonsignificant (right panel) overall OR frequentist estimates compared with Bayesian estimates (half-normal heterogeneity parameter ($\tau$)). Due to scaling requirements, estimates were omitted from (i) Mei et al meta-analysis [13] (frequentist estimate: 1.163(0.327, 5.71); Bayesian: 5.18(1.14, 36.08) and (ii) Poirier et al [14], upper limits of the IVhet OR were limited to 3.5 (estimates: 2.800(0.567, 13.822)


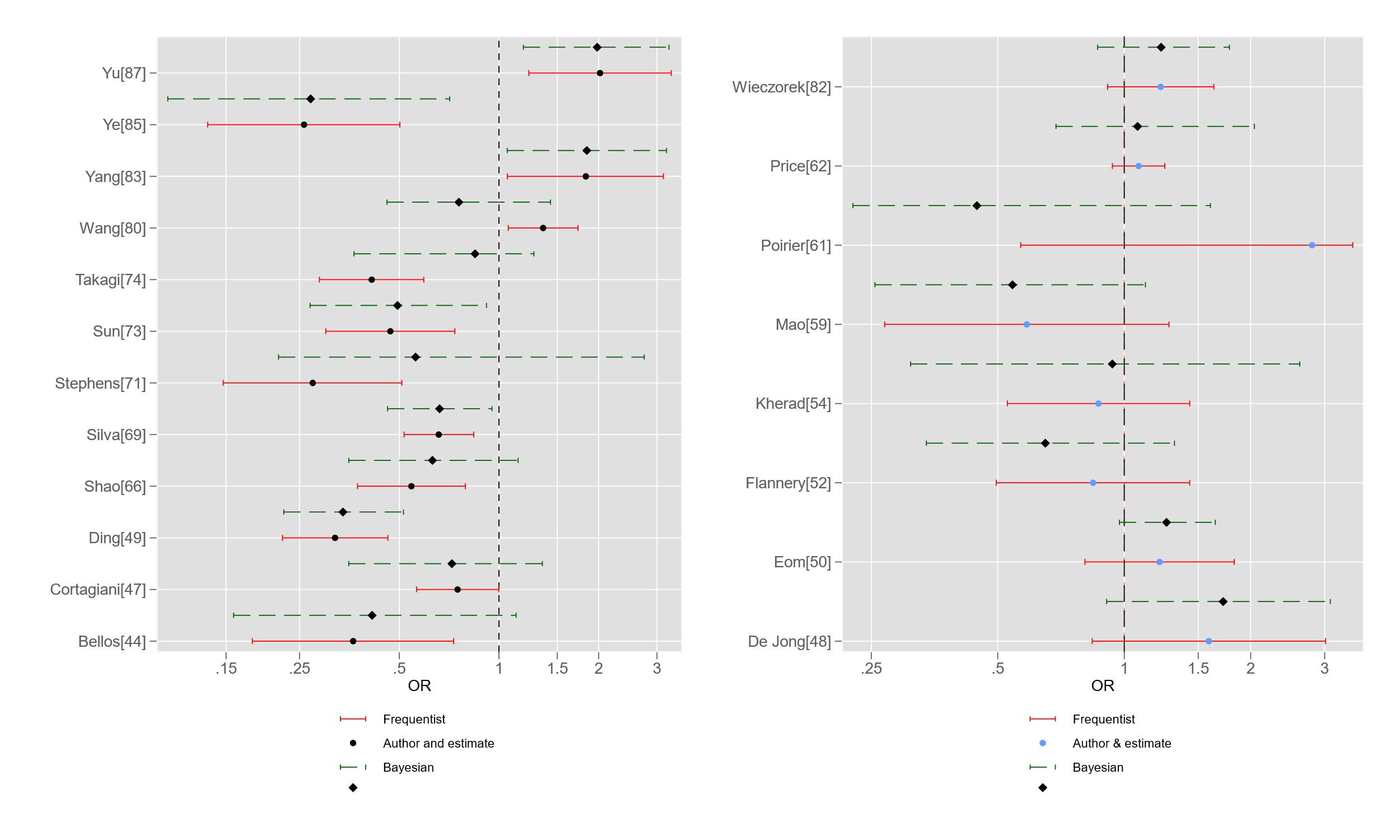


In the OR metric (Figure S3), for significant IVhet estimates (CI not spanning the null), 6 Bayesian CrI spanned the null. For non-significant DSL estimates (CI spanning the null), all Bayesian estimates were consistent. Note must be made of the meta-analysis of Poirier et al [14]; here the RCT point estimates were consistently favourable to intervention and the RCT subgroup effect was 0.20(0.09, 0.44) with I^2^ = 0.0%). There was marked heterogeneity of the NRS sub-group (I^2^ = 88%) and highly significant between-group heterogeneoity (p=0.001). The weight of a single study in the NRS sub-group (NRS effect size: 3.07(0.7, 13.42)), with total patient number 30,703 (effect size: 4.46(3.75, 5.30)) dominated at 67.57%.

Figure S4. Author (frequentist: IVhet) and Bayesian estimates as couplets for RR metric with X-axis on the log scale. Significant (left panel) and nonsignificant (right panel) overall RR frequentist estimates compared with Bayesian estimates (half-Cauchy heterogeneity parameter ($\tau$)).


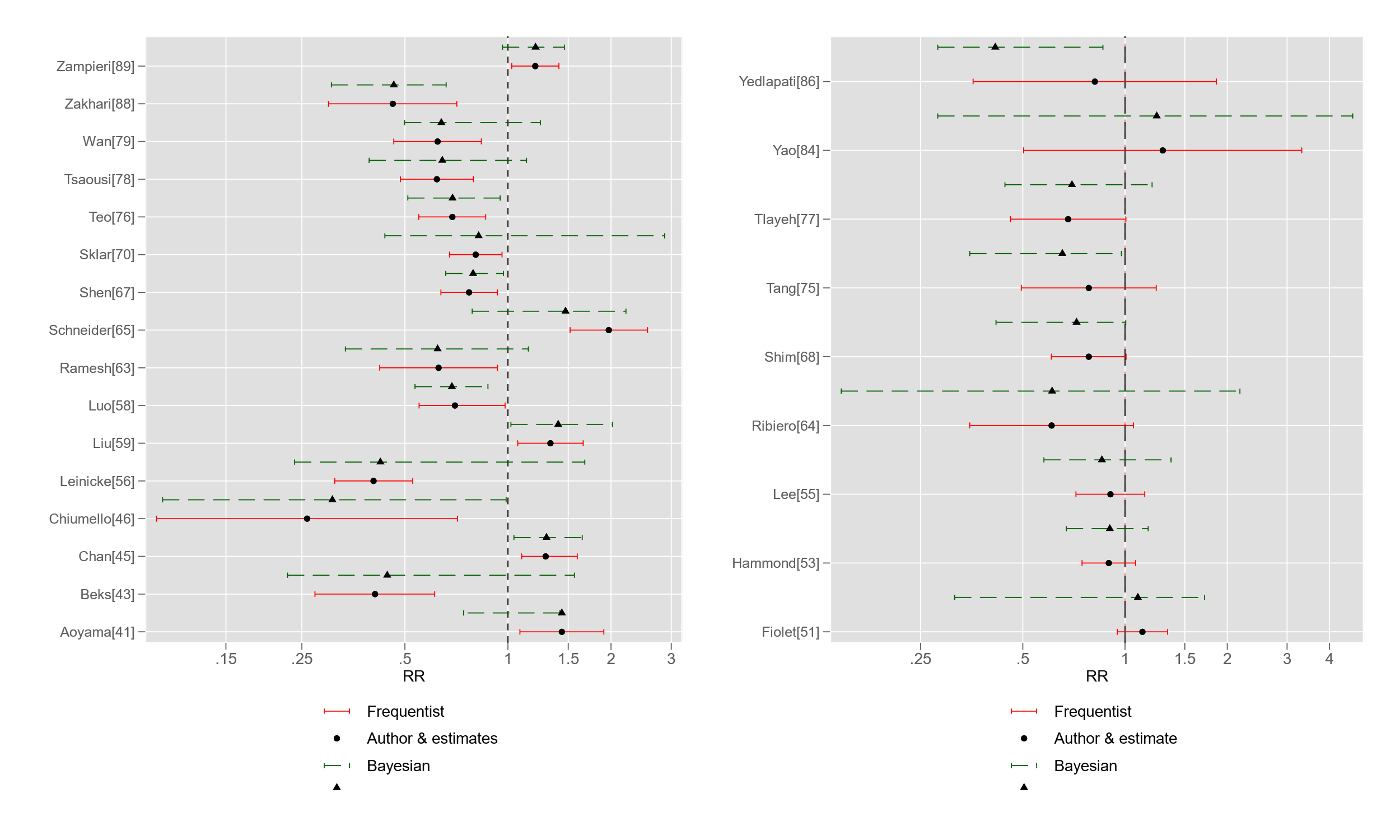


In the RR metric (Figure S4), for significant IVhet estimates (CI not spanning the null), 9 Bayesian CrI spanned the null. For non-significant DSL estimates (CI spanning the null), 1 Bayesian estimates did not span the null.

Table S1. Heterogeneity variance estimates ($\tau^{2}$) for the meta-analytic cohort considered. Generated using the DerSimonian-Laird (DSL) estimator using the user-written Stata module “metan” [7].

Author Author metric Ref. DSL Obs τ^2^ RCT τ^2^ Overall τ^2^

Akingboye OR 42 0.398 0.000 0.225

Aoyama RR 43 0.087 0.000 0.060

Barakakis OR 44 0.000 0.000 0.000

Beks RR 45 0.000 0.000 0.000

Bellos OR 46 0.000 0.000 0.000

Chan RR 47 0.051 0.000 0.011

Chiumello RR 48 0.000 0.000 0.000

Cortegiani OR 49 0.027 0.000 0.009

De Jong OR 50 0.592 0.680 0.511

Ding OR 51 0.148 0.755 0.153

Eom OR 52 0.327 0.115 0.248

Fiolet RR 53 0.000 0.000 0.000

Flannery OR 54 0.261 0.000 0.211

Hammond RR 55 0.000 0.152 0.021

Kherad OR 56 0.011 2.978 0.064

Lee RR 57 0.000 0.073 0.500

Leinicke* RR 58 0.033 0.000 0.029

Liu RR 59 0.040 0.000 0.028

Luo RR 60 0.000 0.256 0.106

Mao OR 61 0.000 0.323 0.354

Mei OR 62 0.520 0.000 0.874

Poirier OR 63 1.120 0.000 1.407

Price** OR 64 0.000 0.000 0.000

Ramesh RR 65 0.032 0.095 0.000

Ribeiro RR 66 0.000 0.479 0.000

Schneider RR 67 0.081 0.000 0.104

Shao OR 68 0.298 0.000 0.263

Shen RR 69 0.053 0.000 0.050

Shim RR 70 0.030 0.000 0.020

Silva OR 71 0.000 0.000 0.000

Sklar RR 72 0.000 0.025 0.020

Stephens OR 73 0.244 0.000 0.271

Sultan* RR 74 0.000 0.000 0.000

Sun OR 75 0.228 0.000 0.129

Tagaki OR 76 0.351 0.000 0.354

Tang RR 77 0.097 0.353 0.109

Teo RR 78 0.000 0.000 0.000

Tlayeh RR 79 0.127 0.029 0.062

Tsaousi RR 80 0.000 0.089 0.013

Wan RR 81 0.000 0.065 0.066

Wang RR 82 0.000 0.028 0.207

Wang OR 83 0.133 0.056 0.136

Wieczorek OR 84 0.338 0.065 0.056

Yang OR 85 0.094 0.822 0.246

Yao RR 86 1.200 0.276 0.523

Ye OR 87 0.000 1.119 1.407

Yedlapati RR 88 0.000 0.000 0.186

Yu OR 89 0.061 0.050 0.038

Zakhari RR 90 1.707 0.007 0.350

Zampieri RR 91 0.000 0.020 0.019

Ref. Paper reference. DSL: DerSimonian-Laird random effects estimator

Obs τ^2^: heterogeneity variance for non-randomised studies (NRS). RCT τ^2^:

heterogeneity variance for randomised controlled trials (RCT). Overall τ^2^:

overall heterogeneity for combined estimate of RCT and NRS.

1. IntHout J, Ioannidis JPA, Borm GF: **The Hartung-Knapp-Sidik-Jonkman method for random effects meta-analysis is straightforward and considerably outperforms the standard DerSimonian-Laird method**. *BMC Med Res Methodol* 2014, **14**.

2. Jackson D, Law M, Rücker G, Schwarzer G: **The Hartung-Knapp modification for random-effects meta-analysis: A useful refinement but are there any residual concerns?** *Stat Med* 2017, **36**(25):3923-3934.

3. Bramley P, López-López JA, Higgins JPT: **Examining how meta-analytic methods perform in the presence of bias: A simulation study**. *Res Synth Methods* 2021, **12**(6):816-830.

4. Doi SAR, Furuya-Kanamori L: **Selecting the best meta-analytic estimator for evidence-based practice: a simulation study**. *International Journal of Evidence-Based Healthcare* 2020, **18**(1):86-94.

5. Doi SAR, Barendregt JJ, Khan S, Thalib L, Williams GM: **Advances in the meta-analysis of heterogeneous clinical trials I: The inverse variance heterogeneity model**. *Contemporary Clinical Trials* 2015, **45**:130-138.

6. Sultan I, Lamba N, Liew A, Doung P, Tewarie I, Amamoo JJ, Gannu L, Chawla S, Doucette J, Cerecedo-Lopez CD *et al*: **The safety and efficacy of steroid treatment for acute spinal cord injury: A Systematic Review and meta-analysis**. *Heliyon* 2020, **6**(2).

7. Fisher D, Harris RJ, Bradburn MJ, Deeks JJ, Harbord RM, Altman DG, Sterne JAC, Higgins J: **metan: fixed- and random-effects meta-analysis; Version 4.07, 15sep2023**. *Available@*[*https://econpapersrepecorg/scripts/searchpf?ft=metan*](https://econpapersrepecorg/scripts/searchpf?ft=metan).

8. Chiumello D, Coppola S, Froio S, Gregoretti C, Consonni D: **Noninvasive ventilation in chest trauma: systematic review and meta-analysis**. *Intensive Care Med* 2013, **39**(7):1171-1180.

9. Ding H, Liao L, Zheng X, Wang Q, Liu Z, Xu G, Li X, Liu L: **Beta-blockers for traumatic brain injury: a systematic review and meta-analysis**. *The journal of trauma and acute care surgery* 2021.

10. Ribeiro RVP, Friedrich JO, Ouzounian M, Yau T, Lee J, Yanagawa B, Canadian Cardiovasc Surg M-A: **Supplemental Cardioplegia During Donor Heart Implantation: A Systematic Review and Meta-Analysis**. *Annals of Thoracic Surgery* 2020, **110**(2):545-552.

11. Takagi H, Umemoto T, Grp A: **A meta-analysis of adjusted observational studies and randomized controlled trials of endovascular versus open surgical repair for ruptured abdominal aortic aneurysm**. *International Angiology* 2016, **35**(6):534-545.

12. Wang C-H, Li C-H, Hsieh R, Fan C-Y, Hsu T-C, Chang W-C, Hsu W-T, Lin Y-Y, Lee C-C: **Proton pump inhibitors therapy and the risk of pneumonia: a systematic review and meta-analysis of randomized controlled trials and observational studies**. *Expert Opinion on Drug Safety* 2019, **18**(3):163-172.

13. Mei H, Wang J, Che H, Wang R, Cai Y: **The clinical efficacy and safety of vancomycin loading dose A systematic review and meta-analysis**. *Medicine* 2019, **98**(43).

14. Poirier Y, Voisine P, Plourde G, Rimac G, Perez AB, Costerousse O, Bertrand OF: **Efficacy and safety of preoperative intra-aortic balloon pump use in patients undergoing cardiac surgery: a systematic review and meta-analysis**. *International Journal of Cardiology* 2016, **207**:67-79.
